# Supplementary material for: Oxidative Stress-Induced HMGB1 Translocation in Myenteric Neurons Contributes to Neuropathy in Colitis
Source: Biomolecules. 2022 Dec 7;12(12):1831. doi: 10.3390/biom12121831 (PMC9776169; doi:10.3390/biom12121831)
Supplement: Supplementary file 1 [file biomolecules-12-01831-s001.zip › biomolecules-2046846-supplementary.pdf]

Supplementary tables

Table S1 ***HMGB1 counts in the myenteric ganglia of Winnie mice***

HMGB1 expression	C57BL/6	*Winnie*
**HMGB1^{+ve} cells/area**		
total	38.5±2.6	30.5±2.3*
neurons	20.1±1.1	11.5±1.6**
non-neuronal	18.4±1.8	19.0±1.2
**Neurons %**		
HMGB1^{-ve}	10.8±3	37.3±8.8*
HMGB1-translocated	0.5±0.3	10.0±4.0*

HMGB1 - High mobility group box-1, HMGB1^{+ve} – HMGB1 expressed in the nucleus, HMGB1^{-ve} – HMGB1 absent from the nucleus. *$P<0.05$, **$P<0.01$ significantly different to C57BL/6. C57BL/6: n=5 mice, *Winnie*: n=7 mice.

Table S2 ***HMGB1 counts in the myenteric plexus of organotypic cultures***

HMGB1 expression	Control	↑O_2	H_2O_2
**$HMGB1^{+ve}$ cells/area**			
neurons	19.5±1.8	12.4±0.7*	11.5±1.9*
non-neuronal	27.7±2.0	24.5±1.7	23.5±2.1
**$HMGB1^{-ve}$ cells/area**			
neurons	2.7±1.3	6.6±2.1*	4.8±1.1*
**HMGB1-translocated cells/area**			
neurons	0.21±0.01	0.92±0.26*	0.71±0.17*
non-neuronal	0.72±0.19	0.87±0.14	1.09±0.29
**Neurons %**			
$HMGB1^{+ve}$	92.8±3.0	76.4±2.5*	69.0±8.3*
$HMGB1^{-ve}$	7.2±3.0	23.6±2.5*	31.1±8.3*
HMGB1-translocated	0.65±0.37	6.05±1.41**	3.76±0.95*

↑O_2 – hyperoxia, H_2O_2, hydrogen peroxide, HMGB1 - High mobility group box-1, $HMGB1^{+ve}$ – HMGB1 expressed in the nucleus, $HMGB1^{-ve}$ – HMGB1 absent from the nucleus. *P<0.05, **P<0.01 significantly different to control. Control: n=8 independent samples, ↑O_2: n=7 independent samples, H_2O_2: n=9 independent samples.

Table S3 ***Correlation between HMGB1 counts and neuronal density in hyperoxia-treated organotypic cultures***

HMGB1 expression	R^2	Relationship to neuronal density	*P* value
**HMGB1^{+ve} cells/area**			
Neurons	0.5874	Increase	0.0022
non-neuronal	0.337	Increase	0.0375
**HMGB1^{-ve} cells/area**			
Neurons	0.4404	Decrease	0.026
**HMGB1-translocated cells/area**			
neurons	0.5794	Decrease	0.004
non-neuronal	0.01006	-	0.7444
**Neurons %**			
HMGB1^{-ve}	0.6063	Decrease	0.0047
HMGB1-translocated	0.6364	Decrease	0.0019

HMGB1 - High mobility group box-1, HMGB1^{+ve} – HMGB1 expressed in the nucleus, HMGB1^{-ve} – HMGB1 absent from the nucleus. Control: n=8 independent samples, ↑O_2: n=7 independent samples.

Table S4 ***Correlation between HMGB1 counts and neuronal density in H_2O_2-treated organotypic cultures***

**HMGB1 expression**	**R^2**	**Relationship to neuronal density**	***P* value**
**$HMGB1^{+ve}$ cells/area**			
neurons	0.6546	Increase	0.0003
non-neuronal	0.1768	-	0.1187
**$HMGB1^{-ve}$ cells/area**			
neurons	0.5551	Decrease	0.0022
**HMGB1-translocated cells/area**			
neurons	0.7457	Decrease	<0.0001
non-neuronal	0.054	-	0.424
**Neurons %**			
$HMGB1^{-ve}$	0.5029	Decrease	0.0045
HMGB1-translocated	0.6684	Decrease	0.0002

H_2O_2, hydrogen peroxide, HMGB1 - High mobility group box-1, $HMGB1^{+ve}$ – HMGB1 expressed in the nucleus, $HMGB1^{-ve}$ – HMGB1 absent from the nucleus. Control: n=8 independent samples, H_2O_2: n=9 independent samples.

Table S5 ***Effects of glycyrrhizic acid treatments on the number of leukocytes in proximity to the myenteric ganglia***

**Location**	**CD45-IR cells/area**		
	**C57BL/6**	***Winnie*-Sham**	***Winnie* GA**
**In ganglia**	0.6±0.2	3.5±0.7***	2.6±0.3**
**Edge of the ganglia**	4.0±0.6	27.1±2.1****	23.2±2.3****
**Outside the ganglia**	3.3±0.7	16.6±2.5****	19.3±1.3****
**Total**	7.8±1.2	47.3±3.2****	44.9±3.6****

GA – glycyrrhizic acid, IR – immunoreactive. **$P<0.01$, ***$P<0.001$, ****$P<0.0001$ significantly different to C57BL/6; n=6 animals/group.
